# A Retrospective Review of CyberKnife Stereotactic Body Radiotherapy for Adrenal Tumors (Primary and Metastatic): Winthrop University Hospital Experience

**DOI:** 10.3389/fonc.2015.00185

**Published:** 2015-08-17

**Authors:** Amishi Desai, Hema Rai, Jonathan Haas, Matthew Witten, Seth Blacksburg, Jeffrey G. Schneider

**Affiliations:** ^1^Department of Hematology and Oncology, Winthrop University Hospital, Mineola, NY, USA

**Keywords:** CyberKnife, adrenal glands, SBRT, metastasis, BED

## Abstract

The adrenal gland is a common site of cancer metastasis. Surgery remains a mainstay of treatment for solitary adrenal metastasis. For patients who cannot undergo surgery, radiation is an alternative option. Stereotactic body radiotherapy (SBRT) is an ablative treatment option allowing larger doses to be delivered over a shorter period of time. In this study, we report on our experience with the use of SBRT to treat adrenal metastases using CyberKnife technology. We retrospectively reviewed the Winthrop University radiation oncology data base to identify 14 patients for whom SBRT was administered to treat malignant adrenal disease. Of the factors examined, the biological equivalent dose (BED) of radiation delivered was found to be the most important predictor of local adrenal tumor control. We conclude that CyberKnife-based SBRT is a safe, non-invasive modality that has broadened the therapeutic options for the treatment of isolated adrenal metastases.

## Introduction

The adrenal gland is a common site of cancer metastasis. In an autopsy series involving 91 patients with metastatic cancer, metastatic spread to the adrenal gland was demonstrated in 30% of patients (1). This propensity for adrenal metastasis, exhibited by many different primary tumor types, is likely a consequence of the adrenal glands’ rich sinusoidal blood supply (2). Lung cancer, the most prevalent form of metastatic cancer, is the most common primary tumor type responsible for adrenal metastases (1, 3). The majority of adrenal metastases are accounted for by lung (35%), gastric (14%), esophageal (12%), and hepatobiliary (10%) primary carcinomas (3).

The adrenal gland is made up of adrenal cortex and medulla. The adrenal cortex consists of the zona glomerulosa, which secretes mineralocorticoids (aldosterone), which regulate sodium and potassium homeostasis. The zona fasciculata secretes glucocorticoids (most importantly, cortisol). The zona reticularis secretes sex steroids (primarily androgens). The adrenal medulla synthesizes and secretes catecholamines, which modulate the body’s sympathetic response to stress. The symptoms and signs of adrenal insufficiency depend upon the rate and extent of loss of adrenal function, whether mineralocorticoid production is preserved, and the degree of stress. The onset of adrenal insufficiency is often very gradual and it may go undetected until an illness or other stress precipitates adrenal crisis.

Clinical manifestations of adrenal insufficiency include weakness, fatigue, anorexia, nausea, vomiting, constipation, hyperpigmentation, hypotension, vitiligo, electrolyte disturbances (hyponatremia, hyperkalemia, hypercalcemia), azotemia, anemia, and eosinophilia. In severe cases, it can lead to shock and death.

Even though surgery still remains a curative option for isolated adrenal metastasis, it can have its own complications like longer hospital stay, perioperative complications, and adrenal insufficiency.

Adrenal gland is located near critical organs, such as stomach, duodenum, small and large bowels, kidneys, spinal cord, liver, one should take into consideration tolerance of these organs in the treatment of adrenal tumors. Rigorous accounting of organ motion is also mandatory to ensure accurate radiotherapy of the adrenal gland. Adrenal function preservation is an added benefit of stereotactic body radiotherapy (SBRT) when compared to surgery.

With modern imaging technologies, the adrenal gland is often found to be a solitary site of metastatic disease. In such cases, surgical resection has often been pursued as definitive therapy. As reported by Lo et al., curative resection of solitary adrenal metastases resulted in overall survival rates of 73% at 1 year and 40% at 2 years (4). Focusing exclusively on non-small cell lung cancer (NSCLC) with solitary adrenal metastases, Tanvetyanon et al. demonstrated 5-year survival rates of 25% following resection of isolated synchronous adrenal metastases and 26% after resection of metachronous adrenal metastases (5). Complication rates ranging from 9 to 20% have been observed in series of patients reported to have undergone adrenalectomy in the management of solitary adrenal metastasis (6–13).

Conventional external beam radiotherapy has been considered an unreliable alternative to surgical resection for the definitive management of solitary adrenal metastases because treatment responses are typically transient and incomplete (6–20). In addition, conventional radiotherapy cannot compensate for tumor motion. In a study of 14 patients with adrenal metastases receiving radiation doses ranging from 16 to 60 Gy, Soejima et al. reported a 6-month survival of 28.6 and 12.5% among the symptomatic group. Despite the poor response, conventional radiation may still prove efficacious for the palliation of pain related to adrenal metastasis (16). However, SBRT has more recently been introduced as a more reliable treatment for the control of the eradication of adrenal metastasis (12). It exploits the more potent radiobiological effect of hypofractionation, larger doses given over a shorter period of time. SBRT precision allows the delivery of ablative doses of radiation to tissue within the planning target volume with small margins to minimize the impact on normal tissue (19). At our institution, the CyberKnife, a robotic-based SBRT delivery system which accounts for intrafraction tumor motion, has been in use since 2005 (19, 20).

One of the advantages of the CyberKnife is the ability to continuously track, in real time the movement of a tumor or target with respiration. Katoh et al. (21) showed that adrenal tumors can move up to 6.1, 11.1, and 7.0 mm in the left-right, craniocaudal, and anterior–posterior directions, respectively. Given the doses used, and the sensitivity of the surrounding anatomy, having the ability to track a tumor that moves during respiration, such as an adrenal tumors or lung tumors is imperative in delivering an ablative dose of radiation without either missing the tumor or damaging surrounding anatomy. This study reports on our experience utilizing this technology to treat malignant adrenal disease.

## Materials and Methods

### Study design

We utilized an Institutional Review Board approved database to retrospectively identify 14 patients for whom SBRT was administered to treat malignant adrenal disease from 2006 to 2011. Charts were reviewed to determine patient characteristics, treatment details, and outcomes. Primary study endpoints were treatment response, duration of response, and survival time measured from the initiation of SBRT. Treatment response was assessed on the basis of routine follow-up imaging studies with CT or PET/CT scan. Local treatment failure was defined as any radiographic progression of adrenal tumor. Distant failure was defined as the development of new metastases or progression of untreated metastases. All patients were treated with SBRT delivered via CyberKnife (Accuray Corporation; Sunnyvale, CA, USA) technology.

All tumors were treated using a CyberKnife robotic linear accelerator. All patients were immobilized using a thermoplastic cast with arms up. One fiducial marker was placed at least 5 days prior under CT guidance by an Interventional Radiologist to account for seed migration. CT imaging was performed using 1.5 mm cuts with and without contrast. At this institution, which as per NCCN guidelines regarding the use of hypofractionated SBRT has appropriate technology, physics, and clinical expertise, all treatments have been given safely and without difficulty. It is important, however, that this expertise be readily available at all times regarding the delivery of this form of treatment given the complexity involved.

**Figure 1:**
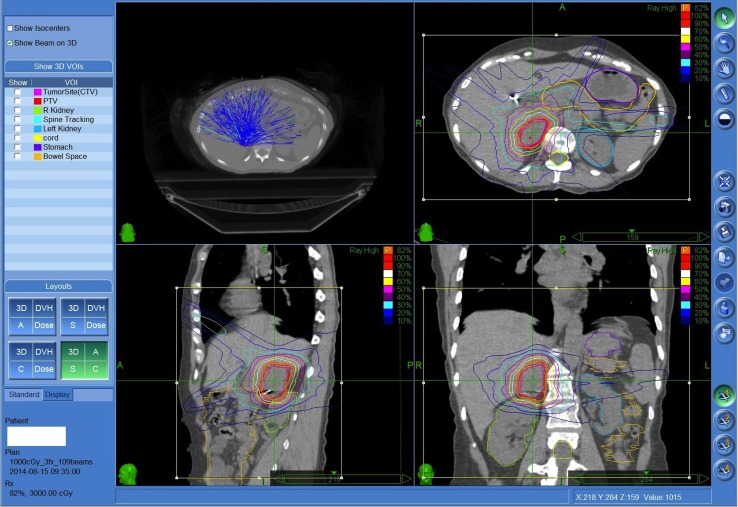
**Fifty-seven-year-old female with metastatic small cell lung cancer with limited biopsy proven painful recurrence in the right adrenal gland despite prior systemic therapy**. Patient was treated to the right adrenal metastasis 3000 cGy in three fractions prescribed to the 82% isodose line. Both kidneys, spinal cord, and regional bowel were contoured.

Planning was performed using Multiplan (Accuray, Inc., Sunnyvale, CA, USA) inverse planning and delivered using the CyberKnife (Accuray, Inc.) with motion and respiratory tracking performed using the Synchrony system (Accuray, Inc.) Only the adrenal tumor was treated rather than the whole gland (Figure 1).

## Results

### Patient characteristics

Patient and tumor characteristics are summarized in Table 1. Median age was 65 years (range, 49–91 years). Primary tumor sites included non-small cell lung (*n* = 6), renal cell (*n* = 2), melanoma (*n* = 1), primary adrenal (*n* = 1), mixed Mullerian (*n* = 1), GE junction (*n* = 1), bladder (*n* = 1), and lymphoma (*n* = 1). Five patients were found to have adrenal involvement at their original cancer diagnosis. For the nine remaining patients, the median interval from first cancer diagnosis to the clinical detection of adrenal metastasis was 14 months (range 8–56). Two patients had pain associated with adrenal metastases in the setting of widespread metastatic disease and received SBRT with palliative intent. Their pain markedly improved after treatment. The other 12 patients had no other sites of active metastasis and received SBRT with definitive intent. These patients did not receive any concurrent chemotherapy while getting CyberKnife.

**Table 1:** **Patient characteristics and outcome**.

Patient	Age	Gender	Primary tumor	Outcome post CK	Time to local failure (months)	Time from CK to death (months)
1	62	M	NSCLC	Stable	7	11
2	91	M	RCC	Regression	+38	NA (still alive)
3	64	M	NSCLC	Stable	2 (until death)	2
4	49	F	NSCLC	Progression	0	NA (still alive)
5	59	M	NSCLC	Stable	5	7
6	63	M	DLBCL	Complete response	+3	NA (still alive)
7	68	M	Melanoma	Regression	4 (until death)	4
8	49	F	RCC	Regression	14 (until death)	14
9	70	F	Adrenocortical carcinoma	Stable	4	11
10	66	F	MMT	NA	NA	9
11	75	F	NSCLC	NA	NA	3
12	71	M	NSCLC	Progression	0	3
13	60	M	GE junction adenocarcinoma	Regression	11 (until death)	11
14	83	M	Urothelial carcinoma	NA	NA	1

*CK, CyberKnife; NSCLC, non-small cell lung cancer; RCC, renal cell carcinoma; DLBCL, diffuse large B cell lymphoma; MMT, mixed Mullerian tumor; GE junction, gastroesophageal*.

### SBRT treatment plans and delivered biological equivalent doses

Individualized SBRT treatment schedules and calculated biological equivalent dose (BED) are shown in Table 2. With the exception of 1 patient with primary bladder cancer receiving 1300 cGy in a single treatment fraction, the remaining 13 patients received a total of either 3 or 5 fractions with each fraction ranging from 500 to 1000 cGy. This heterogeneity in treatment delivery led to a wide range of delivered BEDs (2990–6000 cGy), assuming an alpha/beta ratio of 10.

**Table 2:** **Delivered SBRT regimens and calculated BEDs**.

Patient	Time	Dose (cGy)	#fx	BED (cGy)	180 cGy Eq
1	0	3000	3	6000	5085
2	56	3000	3	6000	5085
3	12	2750	5	4263	3612
4	12	2500	5	3750	3178
5	14	2100	3	3570	3025
6	0	2500	5	3750	3178
7	31	2400	3	4320	3661
8	24	3000	3	6000	5085
9	0	2000	5	2800	2373
10	8	2500	5	3750	3178
11	12	2400	3	4320	3661
12	13	2500	5	3750	3178
13	16	2400	3	4320	3661
14	19	1300	1	2990	2534

### Local adrenal tumor control

There was considerable variation in calculated BEDs (range 4667–13,000 cGy). BED was the most important predictor of local adrenal tumor control. According to best adrenal tumor response, mean BEDs were 10,053 cGy for radiographic regression of disease (*n* = 5); 8115 cGy for stable disease (*n* = 4), and 6667 cGy for progression of disease (*n* = 2), *p* = 0.047. No adrenal metastases resulting from a solid tumor responded to SBRT with BED < 8800 cGy and no patient experienced initial adrenal progression following SBRT with BED > 6667 cGy. Duration of adrenal tumor control also correlated with calculated mean BED, which was 9676 cGy for *never* locally failing (*n* = 6) and 7600 cGy for ever locally failing tumors (*n* = 5). However, eventual local treatment failure was seen in one of three patients receiving even the highest calculated BED (13,000 cGy).

### Toxicity

No patient developed renal or adrenal insufficiency and there were no bowel or spinal cord injuries.

### Literature review

Several groups have previously reported on their experiences with SBRT for the definitive treatment of adrenal oligometastases with conflicting results as summarized in Table 3. For example, Casamassima et al. at the University of Florence reported an impressive 90% local control rate at 2 years (22), whereas Chawla et al. at the University of Rochester reported only a 55% 1 year local control rate (23). These differences may be explained by differences in SBRT dosing and fractionation accounting for significant differences in the prescribed BEDs with maximum delivered BED of 13,730 cGy (36 Gy in 3 fractions) in the Florence series, but just between 2240 cGy (16 Gy in 4 fractions) and 7500 cGy (50 Gy in 10 fractions) in the Rochester cohort (22). Other series have suggested that BEDs > 10,000 cGy are required to achieve optimal local control (24, 25).

**Table 3:** **Characteristics of previous studies using SBRT to treat adrenal metastases**.

Reference/recruitment/country	No. of patients	Radiation dose (median)	Outcome 1 year OS, LC, DC
Chawla et al. (23)/2001–2007/USA	30	400 cGy × 10 fx	44%
			55%
			13%
Katoh et al.* (21)/2004–2006/Japan	9	600 cGy × 8 fx	78%
			100%
Casamassima et al. (22)/2002–2009/Italy	48	1200 cGy × 3 fx	39.7%
			90%
			9%
Holy et al. (26)/2002–2009/Germany	18	720 cGy × 5 fx	23 months
			77%
Torok et al. (27)/2002–2009/USA	7	1700 cGy in 1 fx (1600 cGy in 1 fx and 2700 cGy in 3 fx)	8 months
			63%

*Katoh reference above includes patients with primary adrenal tumors and metastases.

In a series from Hokkaido University, 9 patients with 10 adrenal lesions were treated with SBRT with a dose of 4800 cGy in 8 fractions. The 1-year overall survival and local control rates were 78 and 100%, respectively (21). In contrast to the other groups, they included primary adrenal tumors and perirenal metastatic lymph nodes. In another study by Holy et al., 18 patients with NSCLC and adrenal metastases treated with definitive SBRT for adrenal metastases from NSCLC, experienced a median progression free survival (PFS) of 4.2 months. PFS was markedly increased to 12 months for 13 patients with isolated adrenal metastases. After a median follow-up of 21 months, 10 of these 13 patients achieved local control and median overall survival was 23 months (26). These results compare favorably to the surgical series of Porte et al. where surgical resection of solitary adrenal metastasis was reported to achieve a median PFS of just 13 months (28).

## Discussion

Historically, surgery has been the mainstay of treatment for isolated adrenal metastases. In 1982, Twomey et al. documented prolonged survival following adrenalectomy in the management of oligometastatic NSCLC (29). Patients with a synchronous metastasis who underwent adrenalectomy had a shorter overall survival than those with metachronous metastasis. Overall, subsequent long-term disease free survival has been observed in approximately 25% of patients undergoing resection of solitary adrenal metastasis (6). Long-term survival after resection of isolated NSCLC adrenal metastases was also demonstrated by Mercier et al. with an overall 5-year survival rate of 23.3 and 38% if the isolated adrenal metastasis occurred 6 months after lung resection (11). In colorectal carcinoma, Katayama et al. reported 5 of 11 patients with adrenal metastases remained alive without signs of recurrence after adrenalectomy with follow-up times ranging from 8 months to 9 years (30). In renal cell carcinoma, patients with solitary adrenal metastases achieved a significant tumor specific survival benefit with a median survival of 68 months compared to patients with additional metastatic sites at the time of surgery (31).

In this report, we have retrospectively reviewed our institutional database to identify 14 patients for whom SBRT was administered to treat malignant adrenal disease from 2006 to 2011. Of the factors examined, BED was the most important predictor of local adrenal tumor control. The duration of adrenal tumor control correlated with calculated mean BED, which was 7600 cGy for local failures vs. 9676 cGy for those who attained local control. This finding is supported by other series, which have suggested the delivery of BEDs > 10,000 cGy to achieve optimal tumor control (24, 25). Our experience with patients treated above and below this threshold (median BED of 8460 cGy) also supports the 10,000 cGy threshold.

We observed an initial tumor control rate of 64% (36% tumor regression plus 28% stable disease) similar to the 78% (22% regression plus 56% stable disease) adrenal tumor control rate reported by Torok et al. (27). These small differences could be explained by different tumor types in these two reports. Notably, our series comprises an admixture of different primary tumor types, whereas Torok et al. included only lung and hepatocellular primary carcinomas. In our series, all patients with stable disease following CyberKnife treatment had primary lung cancers. Notably, Torok et al. study population predominantly comprised patients with lung primaries; this could explain the discrepancy in their higher initial response and difference in patients with stable disease. The transient nature of prolonged control from metastatic lung primaries was demonstrated in both studies.

In addition, we also looked at tumor histology as it predicted for treatment outcome. We had an admixture of different primaries, but the majority was six patients with NSCLC: four adenocarcinoma, one squamous cell carcinoma, and one high-grade sarcoma. The latter two patients progressed after treatment, which could be attributable to aggressive histology. The three adenocarcinomas remained stable with two eventually progressing locally at 5 and 7 months and one remained stable until failing distantly at 2 months. The fourth patient opted for palliative care, so no post treatment scans were obtained. Other primaries fared better with regression in the size of the lesion noted in renal carcinoma, GE junction adenocarcinoma, and melanoma. Three of these patients eventually succumbed to their disease from distant failure. Complete response was documented for diffuse large B cell lymphoma.

While surgical resection remains a suitable option for patients with isolated adrenal metastases who are able to undergo that approach, CyberKnife-based SBRT is a safe, non-invasive alternative modality that has broadened the therapeutic options for the attainment of palliation and local control of this historically difficult-to-manage patient cohort. When utilized in this setting, we recommend targeting a BED of at least 10,000 cGy. We also encourage consideration of this approach in all patients with solitary adrenal metastasis who cannot or will not undergo surgical resection. Larger series and increased follow-up times will be required in the future evaluation of this treatment.

## Conflict of Interest Statement

The authors declare that the research was conducted in the absence of any commercial or financial relationships that could be construed as a potential conflict of interest.
